# Supplementary material for: Single Lead Epidural Spinal Cord Stimulation Targeted Trunk Control and Standing in Complete Paraplegia
Source: J Clin Med. 2022 Aug 30;11(17):5120. doi: 10.3390/jcm11175120 (PMC9457264; doi:10.3390/jcm11175120)
Supplement: Supplementary file 1 [file jcm-11-05120-s001.zip › Suppl. Figure S1.pdf]

INTERNATIONAL STANDARDS FOR NEUROLOGICAL CLASSIFICATION OF SPINAL CORD INJURY (ISNCSCI)

AMERICAN SPINAL INJURY ASSOCIATION — ISCOS INTERNATIONAL SPINAL CORD SOCIETY

Patient Name 001 Date/Time of Exam 02/25/2022

Examiner Name ______ Signature ______

RIGHT / LEFT

RIGHT Motor Key Muscles	Level	Light Touch (LTR)	Pin Prick (PPR)	Light Touch (LTL)	Pin Prick (PPL)	LEFT Motor Key Muscles	
	C2	2	2	2	2		
	C3	2	2	2	2		
	C4	2	2	2	2		
5	C5	2	2	2	2	5	*Elbow flexors*
5	C6	2	2	2	2	5	*Wrist extensors*
5	C7	2	2	2	2	5	*Elbow extensors*
5	C8	2	2	2	2	5	*Finger flexors*
5	T1	2	2	2	2	5	*Finger abductors (little finger)*
	T2	2	2	2	2		
	T3	2	2	2	2		
	T4	1	1	1	1		
	T5	1	1	1	1		
	T6	1	1	1	1		
	T7	1	1	1	1		
	T8	0	0	0	0		
	T9	0	0	0	0		
	T10	0	0	0	0		
	T11	0	0	0	0		
	T12	0	0	0	0		
	L1	0	0	0	0		
0	L2	0	0	0	0	0	*Hip flexors*
0	L3	0	0	0	0	0	*Knee extensors*
0	L4	0	0	0	0	0	*Ankle dorsiflexors*
0	L5	0	0	0	0	0	*Long toe extensors*
0	S1	0	0	0	0	0	*Ankle plantar flexors*
	S2	0	0	0	0		
	S3	0	0	0	0		
	S4-5	0	0	0	0		
**RIGHT TOTALS** 25		24	24	24	24	25	**LEFT TOTALS**
(MAXIMUM) (50)		(56)	(56)	(56)	(56)	(50)	(MAXIMUM)

UER (Upper Extremity Right) — **UEL** (Upper Extremity Left)

LER (Lower Extremity Right) — **LEL** (Lower Extremity Left)

Comments *(Non-key Muscle? Reason for NT? Pain? Non-SCI condition?):*

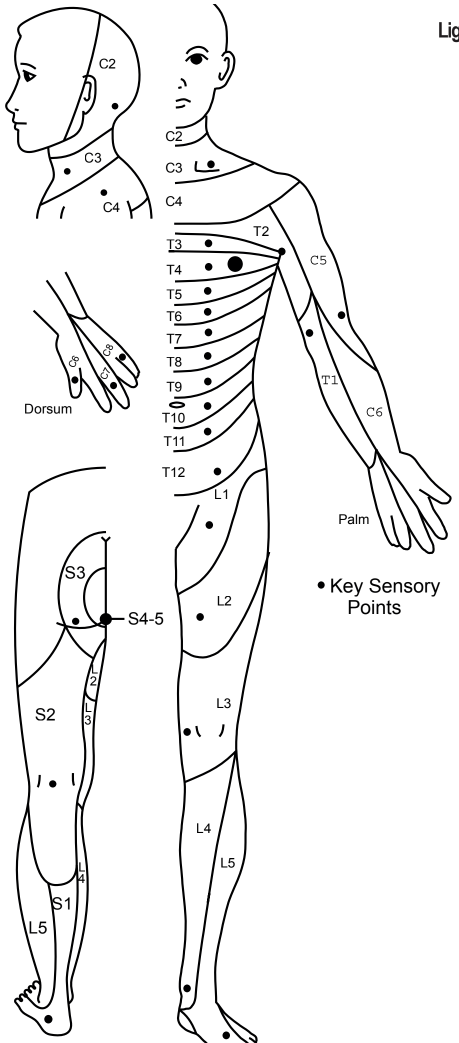

(VAC) Voluntary Anal Contraction (Yes/No) No

(DAP) Deep Anal Pressure (Yes/No) No

MOTOR (SCORING ON REVERSE SIDE)

0 = Total paralysis
1 = Palpable or visible contraction
2 = Active movement, gravity eliminated
3 = Active movement, against gravity
4 = Active movement, against some resistance
5 = Active movement, against full resistance
NT = Not testable
0, ..., 4*, NT* = Non-SCI condition present*

SENSORY (SCORING ON REVERSE SIDE)

0 = Absent *NT = Not testable*
1 = Altered *0*, 1*, NT* = Non-SCI condition present*
2 = Normal

MOTOR SUBSCORES

UER 25 + UEL 25 = *UEMS TOTAL* 50 — MAX (25) (25) (50)

LER 0 + LEL 0 = *LEMS TOTAL* 0 — MAX (25) (25) (50)

SENSORY SUBSCORES

LTR 24 + LTL 24 = *LT TOTAL* 48 — MAX (56) (56) (112)

PPR 24 + PPL 24 = *PP TOTAL* 48 — MAX (56) (56) (112)

NEUROLOGICAL LEVELS

Steps 1- 6 for classification as on reverse

	R	L
1. SENSORY	T3	T3
2. MOTOR	T3	T3

3. NEUROLOGICAL LEVEL OF INJURY (NLI): T3

4. COMPLETE OR INCOMPLETE? C
Incomplete = Any sensory or motor function in S4-5

5. ASIA IMPAIRMENT SCALE (AIS): A

6. ZONE OF PARTIAL PRESERVATION (In injuries with absent motor OR sensory function in S4-5 only)
Most caudal levels with any innervation

	R	L
SENSORY	T7	T7
MOTOR	T3	T3

This form may be copied freely but should not be altered without permission from the American Spinal Injury Association.

Muscle Function Grading

0 = Total paralysis

1 = Palpable or visible contraction

2 = Active movement, full range of motion (ROM) with gravity eliminated

3 = Active movement, full ROM against gravity

4 = Active movement, full ROM against gravity and moderate resistance in a muscle specific position

5 = (Normal) active movement, full ROM against gravity and full resistance in a functional muscle position expected from an otherwise unimpaired person

NT = Not testable (i.e. due to immobilization, severe pain such that the patient cannot be graded, amputation of limb, or contracture of > 50% of the normal ROM)

0*, ..., 4*, NT* = Non-SCI condition present [a]

Sensory Grading

0 = Absent **1** = Altered, either decreased/impaired sensation or hypersensitivity

2 = Normal **NT** = Not testable

0*, 1*, NT* = Non-SCI condition present [a]

[a] Note: Abnormal motor and sensory scores should be tagged with a "*" to indicate an impairment due to a non-SCI condition. The non-SCI condition should be explained in the comments box together with information about how the score is rated for classification purposes (at least normal / not normal for classification).

When to Test Non-Key Muscles:

In a patient with an apparent AIS B classification, non-key muscle functions more than 3 levels below the motor level on each side should be tested to most accurately classify the injury (differentiate between AIS B and C).

Movement	Root level
**Shoulder:** Flexion, extension, adbuction, adduction, internal and external rotation<br>**Elbow:** Supination	C5
**Elbow:** Pronation<br>**Wrist:** Flexion	C6
**Finger:** Flexion at proximal joint, extension<br>**Thumb:** Flexion, extension and abduction in plane of thumb	C7
**Finger:** Flexion at MCP joint<br>**Thumb:** Opposition, adduction and abduction perpendicular to palm	C8
**Finger:** Abduction of the index finger	T1
**Hip:** Adduction	L2
**Hip:** External rotation	L3
**Hip:** Extension, abduction, internal rotation<br>**Knee:** Flexion<br>**Ankle:** Inversion and eversion<br>**Toe:** MP and IP extension	L4
**Hallux and Toe:** DIP and PIP flexion and abduction	L5
**Hallux:** Adduction	S1

ASIA Impairment Scale (AIS)

A = Complete. No sensory or motor function is preserved in the sacral segments S4-5.

B = Sensory Incomplete. Sensory but not motor function is preserved below the neurological level and includes the sacral segments S4-5 (light touch or pin prick at S4-5 or deep anal pressure) AND no motor function is preserved more than three levels below the motor level on either side of the body.

C = Motor Incomplete. Motor function is preserved at the most caudal sacral segments for voluntary anal contraction (VAC) OR the patient meets the criteria for sensory incomplete status (sensory function preserved at the most caudal sacral segments (S4-S5) by LT, PP or DAP), and has some sparing of motor function more than three levels below the ipsilateral motor level on either side of the body.
(This includes key or non-key muscle functions to determine motor incomplete status.) For AIS C – less than half of key muscle functions below the single NLI have a muscle grade ≥ 3.

D = Motor Incomplete. Motor incomplete status as defined above, with at least half (half or more) of key muscle functions below the single NLI having a muscle grade ≥ 3.

E = Normal. If sensation and motor function as tested with the ISNCSCI are graded as normal in all segments, and the patient had prior deficits, then the AIS grade is E. Someone without an initial SCI does not receive an AIS grade.

Using ND: To document the sensory, motor and NLI levels, the ASIA Impairment Scale grade, and/or the zone of partial preservation (ZPP) when they are unable to be determined based on the examination results.

AMERICAN SPINAL INJURY ASSOCIATION

INTERNATIONAL STANDARDS FOR NEUROLOGICAL CLASSIFICATION OF SPINAL CORD INJURY

ISCOS
INTERNATIONAL SPINAL CORD SOCIETY

Steps in Classification

The following order is recommended for determining the classification of individuals with SCI.

1. Determine sensory levels for right and left sides.
The sensory level is the most caudal, intact dermatome for both pin prick and light touch sensation.

2. Determine motor levels for right and left sides.
Defined by the lowest key muscle function that has a grade of at least 3 (on supine testing), providing the key muscle functions represented by segments above that level are judged to be intact (graded as a 5).
Note: in regions where there is no myotome to test, the motor level is presumed to be the same as the sensory level, if testable motor function above that level is also normal.

3. Determine the neurological level of injury (NLI)
This refers to the most caudal segment of the cord with intact sensation and antigravity (3 or more) muscle function strength, provided that there is normal (intact) sensory and motor function rostrally respectively.
The NLI is the most cephalad of the sensory and motor levels determined in steps 1 and 2.

4. Determine whether the injury is Complete or Incomplete.
(i.e. absence or presence of sacral sparing)
*If voluntary anal contraction = **No** AND all S4-5 sensory scores = **0***
*AND deep anal pressure = **No**, then injury is **Complete.***
*Otherwise, injury is **Incomplete**.*

5. Determine ASIA Impairment Scale (AIS) Grade:

Is injury Complete? If YES, AIS=A

NO ↓

Is injury Motor Complete? If YES, AIS=B

NO ↓ (No=voluntary anal contraction OR motor function more than three levels below the motor level on a given side, if the patient has sensory incomplete calssification)

Are at least half (half or more) of the key muscles below the neurological level of injury graded 3 or better?

NO ↓ **AIS=C**

YES ↓ **AIS=D**

If sensation and motor function is normal in all segments, AIS=E
Note: AIS E is used in follow-up testing when an individual with a documented SCI has recovered normal function. If at initial testing no deficits are found, the individual is neurologically intact: the ASIA Impairment Scale does not apply.

6. Determine the zone of partial preservation (ZPP)
The ZPP is used only in injuries with absent motor (no VAC) OR sensory function (no DAP and no LT and no PP sensation) in the lowest sacral segments S4-5, and refers to those dermatomes and myotomes caudal to the sensory and motor levels that remain partially innervated. With sacral sparing of sensory function, the sensory ZPP is not applicable and therefore "NA" is recorded in the block of the worksheet. Accordingly, if VAC is present, the motor ZPP is not applicable and is noted as "NA".
